# The cross-sectional relationship between body dysmorphic disorder and perfectionism: a meta-analysis

**DOI:** 10.1186/s12888-026-08044-7

**Published:** 2026-04-21

**Authors:** Rafaela Stranz, Frederic Maas genannt Bermpohl, Katharina Bosbach, Anja Grocholewski, Alexandra Martin, Andrea S. Hartmann

**Affiliations:** 1 Department of Clinical Psychology and Psychotherapy of Childhood and Adolescence, University of Konstanz, Konstanz, Germany; 2 Department of Clinical Psychology and Psychotherapy, Faculty of Human and Social Sciences, University of Wuppertal, Wuppertal, Germany; 3 Department of Psychology, Technische Universität Braunschweig, Braunschweig, Germany

**Keywords:** Meta-analysis, Body dysmorphic disorder, Body dysmorphic symptoms, Muscle dysmorphia, Perfectionism, Perfectionistic self-presentation, Personality traits

## Abstract

Perfectionism has been theoretically implicated in the development and maintenance of body dysmorphic disorder (BDD), but a quantitative synthesis of its cross-sectional relationship with BDD symptom severity is lacking. The present meta-analysis therefore examines the strength and consistency of this relationship across existing studies. A random-effects meta-analysis of 34 studies comprising a total of 13 107 participants was conducted. Studies were included if they assessed BDD symptoms and perfectionism using validated instruments. Subgroup analyses were used to examine perfectionism dimensions, sample characteristics, and diagnostic subtypes. Partial correlations controlling for depression were examined. The results revealed a moderate and statistically significant correlation between BDD symptoms and perfectionism (*r* = .32, 95% *CI* [0.28, 0.36]). The association remained stable across age groups, sample types, and genders, and was comparable in individuals with muscle dysmorphia (*r* = .34). A separate meta-analysis focusing on individuals with elevated BDD symptoms yielded a comparable effect size (*r* = .35, 95% *CI* [0.22, 0.47]). Controlling for depressive symptoms attenuated the relationship, indicating that part of the association may be attributable to shared depressive symptomatology. Significant heterogeneity (*I²* = 81%) and evidence of publication bias were detected, although the findings were comparable across several sensitivity analyses. The results highlight the relevance of both perfectionistic concerns and perfectionistic strivings as correlates of BDD-related distress and support its consideration within transdiagnostic models. The protocol for this meta-analysis was pre-registered on PROSPERO (Preregistration ID: CRD42024551099).

**Supplementary Information:**

The online version contains supplementary material available at 10.1186/s12888-026-08044-7.

## Introduction

Body dysmorphic disorder (BDD) is characterized by a persistent preoccupation with perceived or minor flaws in physical appearance (e.g., nose is too big, hair too thin, body not muscular enough; the latter in the case of the subtype muscle dysmorphia (MD)), which are typically not observable or appear slight to others. Moreover, individuals with BDD show time-consuming repetitive behaviors such as mirror checking, grooming, or comparing their appearance to others [3, 51], and frequently experience severe distress, functional impairment, and reduced quality of life [38] as well as elevated rates of suicidal ideation and suicide attempts [4, 51]. Epidemiological studies estimate the lifetime prevalence to be approximately 2–3% in the general population, with higher rates observed in clinical samples [67]. Body dysmorphic concern refers to subclinical symptoms of appearance-related distress that do not fulfil the diagnostic criteria for BDD [18].

Cognitive-behavioral models of BDD emphasize rigid appearance-related standards, heightened self-focused attention, and negatively biased evaluation of perceived flaws as central cognitive processes in symptom expression, alongside repetitive and avoidant behaviors such as mirror checking, appearance comparison, reassurance seeking, camouflaging, and avoidance as key maintaining mechanisms [66, 69]. These processes conceptually align particularly with perfectionistic concerns, insofar as intolerance of perceived imperfections may increase the salience of minor appearance deviations and co-occur with characteristic checking, comparison, and avoidance behaviors [67]. In addition, perfectionism has been proposed as a potential vulnerability factor in etiological models of BDD, with rigid personal standards, fear of mistakes, and heightened self-criticism assumed to be associated with increased risk for symptom development and to contribute to symptom maintenance [69]. In the case of MD, perfectionistic tendencies may be oriented toward muscularity, strength, and physique-related ideals, which differ in content from appearance concerns more commonly observed in other BDD presentations [11].

Consistent with these theoretical considerations, empirical research has increasingly examined perfectionism in relation to BDD, although this literature remains comparatively limited, with few recent studies addressing this relationship directly. Across studies, individuals with BDD report elevated standards of attractiveness and heightened self-evaluative concerns, alongside increased self-focused attention, hypervigilance, and difficulties in emotion regulation [10, 39]. Empirical findings further indicate that individuals with BDD consistently report higher levels of perfectionism compared to unaffected controls [10, 22, 30, 34, 35, 39, 60]. Emerging evidence further suggests that associations between perfectionism and BDD symptom severity are not uniform across facets, with some dimensions appearing more closely related to symptom severity than others and therefore discussed as theoretically relevant correlates within existing models [9, 35, 39].

To contextualize these associations, it is necessary to outline how perfectionism is conceptualized and operationalized in contemporary research. Perfectionism is a multifaceted personality trait characterized by rigid standards and an aspiration for flawlessness [20], typically accompanied by critical self-evaluation and feelings of inadequacy [9]. Contemporary perfectionism research distinguishes between two higher-order dimensions: perfectionistic concerns (e.g., fear of mistakes, doubts about actions, self-critical evaluation) and perfectionistic strivings (e.g., setting and pursuing high personal standards), which have shown differential associations with psychopathology in prior meta-analyses, with perfectionistic concerns demonstrating consistently moderate associations across disorders, whereas perfectionistic strivings show smaller and more variable associations [12, 43].

Several instruments operationalize perfectionistic concerns and strivings, including measures such as the *Frost Multidimensional Perfectionism Scale* (FMPS; [23]) and the *Multidimensional Perfectionism Scale* (MPS; [31]). Consistent with this conceptual overlap, studies have linked BDD symptom severity to concern-related facets of perfectionism (e.g., fear of mistakes and doubt/uncertainty), whereas striving-related facets reflecting high personal standards show weaker or less consistent associations, and facets less directly tied to evaluative threat (e.g., organization) tend to show weaker or no associations [26, 30]. While perfectionistic concerns and BDD-related cognitions are conceptually related, they remain theoretically distinct, and this overlap may inflate associations in self-report studies.

Beyond BDD, perfectionism is consistently associated with a range of mental health symptoms across various disorders, including depression, eating disorders (EDs), and obsessive-compulsive disorder (OCD; [12, 43]). For example, in EDs, body dissatisfaction and idealized appearance standards are prominent [33, 38, 56], and perfectionistic concerns have been linked to body checking, drive for thinness, and disordered eating, alongside greater appearance-contingent self-worth [29, 30, 33]. Given its established associations with depression, ED, and OCD-related processes (e.g., negative self-evaluation, doubt, checking, intolerance of imperfection), this raises the question of whether perfectionism contributes to BDD symptom severity via BDD-specific mechanisms emphasized in etiological models or primarily reflects broader transdiagnostic distress and shared vulnerability.

Taken together, these findings underscore the complex interplay between perfectionism and psychopathological symptoms, highlighting the need for comprehensive approaches to their investigation. Although several studies have demonstrated an association between BDD and perfectionism, a systematic and quantitative synthesis is lacking. Previous research has not clarified the overall strength of this association or systematically examined perfectionism dimensions within a theoretically grounded framework. Although perfectionism has been examined in relation to broader psychopathology and related disorders [12, 43, 52] as well as BDD in relation to OCD [46], no quantitative synthesis has specifically focused on the association between perfectionism and BDD symptom severity. Given the distinct, appearance-focused phenomenology of BDD, a disorder-specific synthesis is warranted. The present meta-analysis therefore provides a quantitative synthesis of this association across general population and clinical samples, clarifying its overall magnitude and informing whether it reflects BDD-specific or broader transdiagnostic processes. Specifically, this meta-analysis aims to (a) estimate the correlation between BDD symptom severity and perfectionism, (b) examine associations of BDD symptom severity with distinct perfectionism dimensions, with a focus on concern- and striving-related facets as operationalized by commonly used FMPS and conceptually equivalent MPS subscales, (c) test variation across sample characteristics (age, gender, sample type), (d) compare associations across diagnostic subtypes (BDD vs. MD), and (e) evaluate whether the association remains statistically evident when controlling for depressive symptoms, which were selected a priori as the most consistently assessed and reported comorbid condition across studies of BDD [64, 67], thereby allowing for meta-analytic adjustment; comparable analyses for other highly comorbid conditions, such as OCD and EDs, were planned but were not feasible due to insufficient reporting.

## Methods

The protocol for this meta-analysis was pre-registered on PROSPERO in June 2024 (Preregistration ID: CRD42024551099). Template extraction forms, extracted datasets, analysis code, and additional materials are available on OSF (osf.io/dk7cn). A PRISMA checklist concerning the documentation of the meta-analysis can be retrieved from the supplement.

### Study selection

The inclusion criteria were defined according to a PICO framework. Given the cross-sectional nature of the included studies, exposure and outcome were conceptualized as correlated constructs rather than as directional effects. Population: individuals across the full severity spectrum of body image concern, from minimal body dissatisfaction to clinically significant BDD; Exposure: perfectionism assessed using validated instruments; and Outcome: BDD symptom severity assessed using self-report questionnaires, structured clinical interviews, or formal diagnoses based on the DSM (American Psychiatric Association, 2013) or ICD (World Health Organization [70]). For example, the included studies applied the *Yale-Brown Obsessive-Compulsive Scale modified for BDD* (BDD-YBOCS) or the *Dysmorphic Concern Questionnaire* (DCQ). Perfectionism measures included self-report questionnaires such as the FMPS, MPS, and the *Perfectionistic Self-Presentation Scale* (PSPS), proving that sufficient evidence of reliability and construct validity was available. If multiple BDD symptom or perfectionism measures were reported, the primary outcome identified by the original authors was selected. A full overview of the included measures is provided in the supplementary material.

The study population encompassed a broad range of participants, including clinical, subclinical, and non-clinical samples (e.g., students, community). If a study included a control group, it was coded accordingly. Correlations within control groups were calculated if sufficient data were available. Studies with various research designs were considered, including (randomized) controlled trials, observational studies (e.g., cohort or case-control designs), and cross-sectional studies. In studies with longitudinal or interventional designs, only baseline (pre-intervention) data were extracted and included in the meta-analysis; when studies included multiple groups (e.g., intervention and control groups), baseline correlations were extracted separately. No restrictions were applied regarding participants’ age or gender. Eligible publications were limited to articles written in English or German.

### Information sources

A comprehensive literature search was conducted from June to December 2024, and an updated search using the same search string was conducted in December 2025. The search strategy encompassed multiple electronic databases (*PubMed*,* Psyndex*,* PsycINFO*,* Web of Science*,* ProQuest).* The search algorithms are provided in the supplement. Additionally, reference lists of all included studies and relevant reviews, as well as forward citations of key articles, were screened to identify additional eligible studies, following standard procedures for systematic reviews. The search was not limited by publication date, allowing for a comprehensive review of the literature.

### Data collection and coding

Duplicate records were removed using ZOTERO (Version 7.0). Titles and abstracts were screened by four student raters working in parallel to identify studies assessing BDD symptoms and perfectionism. Each record was independently assessed by two raters, and discrepancies were resolved by the first author, who served as a fifth reviewer. The same procedure was applied at the full-text stage. Reasons for exclusion were documented.

Data extraction was conducted by two independent raters using a standardized coding form. Extracted variables included study and sample characteristics, measures of BDD symptoms and perfectionism, and effect sizes. Perfectionism was primarily extracted as a total score. Although preregistered analyses focused on overall perfectionism and available subscale-level associations, findings were interpreted within the higher-order distinction between perfectionistic concerns and strivings to facilitate theoretical integration. Dimension-specific synthesis focused on FMPS-based subscales that capture theoretically relevant perfectionism facets and were reported with sufficient frequency. Consistent with the higher-order framework, *Concern over Mistakes* and *Doubts about Actions* indexed perfectionistic concerns, whereas *Personal Standards* was classified as an indicator of perfectionistic strivings. Additional FMPS-based subscales (e.g., Parental Expectations, Organization) were coded when available but were reported too infrequently for synthesis; dimensional analyses focused a priori on facets aligned with evaluative threat and rigid standards in BDD, with further analyses contingent on data availability. For studies using perfectionism instruments other than the FMPS/MPS, reported subscales were documented during data extraction but not synthesized at the subscale level; when only subscale-level correlations were available, weighted means were used to derive an overall perfectionism effect size. Interrater reliability was calculated for the data extraction process (κ = 0.66 for categorical data; ICC = 0.74 for continuous data). For further details of the coding procedure, see supplement.

### Methodological quality assessment

To grade the quality of evidence, the four raters assessed the included studies using an adapted Joanna Briggs Institute risk of bias tool [6], with the studies categorized as follows: 15 or more “yes” ratings indicated a low risk of bias, 10 to 14 a moderate risk, and fewer than 10 a high risk. Given the large number of items and the conservative nature of dichotomous coding, this classification was intended to provide a general overview of methodological quality while allowing for some variability across study designs. The tool was used to systematically evaluate methodological quality across domains (e.g., sample selection, adequacy of sample description, replicability, reporting transparency). Any discrepancies were resolved through discussion, with the first author being consulted if consensus could not be reached. In the case of missing effect sizes, we contacted the respective study authors.

### Data analysis

The meta-analysis was conducted in accordance with established methodological guidelines [8, 50]. The data analysis was conducted in *R* (version 4.4.3; R Core Team, [54]) using the metafor package [68]. Effect sizes (Pearson’s *r*) were transformed into Fisher’s z-values using the *escalc()* function, synthesized using random-effects meta-analytic models via *rma()* with the restricted maxi-mum likelihood (REML) estimator, and subsequently back-transformed for interpretation. A meta-analysis was performed to quantify the cross-sectional association between BDD symptom severity and perfectionism. Pooled effect sizes and heterogeneity statistics (e.g., *I*², *τ*²) were calculated, and final estimates were visualized using a forest plot, delineating study-specific effects and global effect magnitude. For further analysis of different perfectionism dimensions, a separate random-effects model with the REML estimator was used following the same procedure.

Subgroup analyses were performed to assess the impact of moderators (age, gender, diagnostic subtype, and clinical status). Regarding age, pooled effect sizes are calculated separately for adult, youth, and mixed-age samples. For the subgroup analysis by gender (female vs. male), only samples consisting exclusively of female or exclusively of male participants were included. In addition, we conducted specific subgroup comparisons between studies assessing general BDD symptoms with those assessing MD symptoms to explore potential differences in effect sizes. Finally, subgroup analyses by clinical status categorized studies into those including participants who met full diagnostic or probable BDD criteria (type 1) and those with subclinical symptoms or elevated body dissatisfaction (type 2). Categorical variables were dummy-coded where appropriate. Subgroup comparisons required a minimum of *k* ≥ 3 studies per group, and statistical significance was set at an alpha level of *α* = 0.05.

To examine whether the association remains after adjusting for depressive symptoms, additional meta-analyses were conducted using partial correlation coefficients where sufficient data were available. Partial correlations were computed within each study based on the intercorrelations among BDD symptoms, perfectionism, and depressive symptoms, following established procedures [40]. These coefficients were Fisher z-transformed and synthesized using random-effects meta-analysis, applying the same analytic pipeline as for zero-order correlations. Comparable adjustments for other theoretically relevant comorbid conditions, such as OCD or EDs, would have been informative, but inconsistent reporting across studies precluded their inclusion in meta-analytic control.

For the assessment of publication bias, we performed funnel plot analyses. In cases of observed asymmetry, Egger’s regression test was conducted to evaluate small-study effects. To assess the robustness of the pooled effect estimates, sensitivity analyses included leave-one-out analyses, funnel plot inspection, and analyses stratified by risk-of-bias categories. In addition, reliability with respect to small-study effects and influence of individual studies was independently evaluated by two raters, with discrepancies resolved through discussion or consultation with a third reviewer.

Deviating from the preregistered plan, we conducted a second, more focused meta-analysis to examine whether the association between BDD symptoms and perfectionism also holds within a diagnostically defined subgroup. This approach of analyzing a diagnostically more homogeneous sample reduced the influence of large numbers of individuals with minimal or no symptoms. Effect sizes were interpreted in accordance with Cohen’s [16] guidelines.

## Results

### Study characteristics

**Fig. 1:**
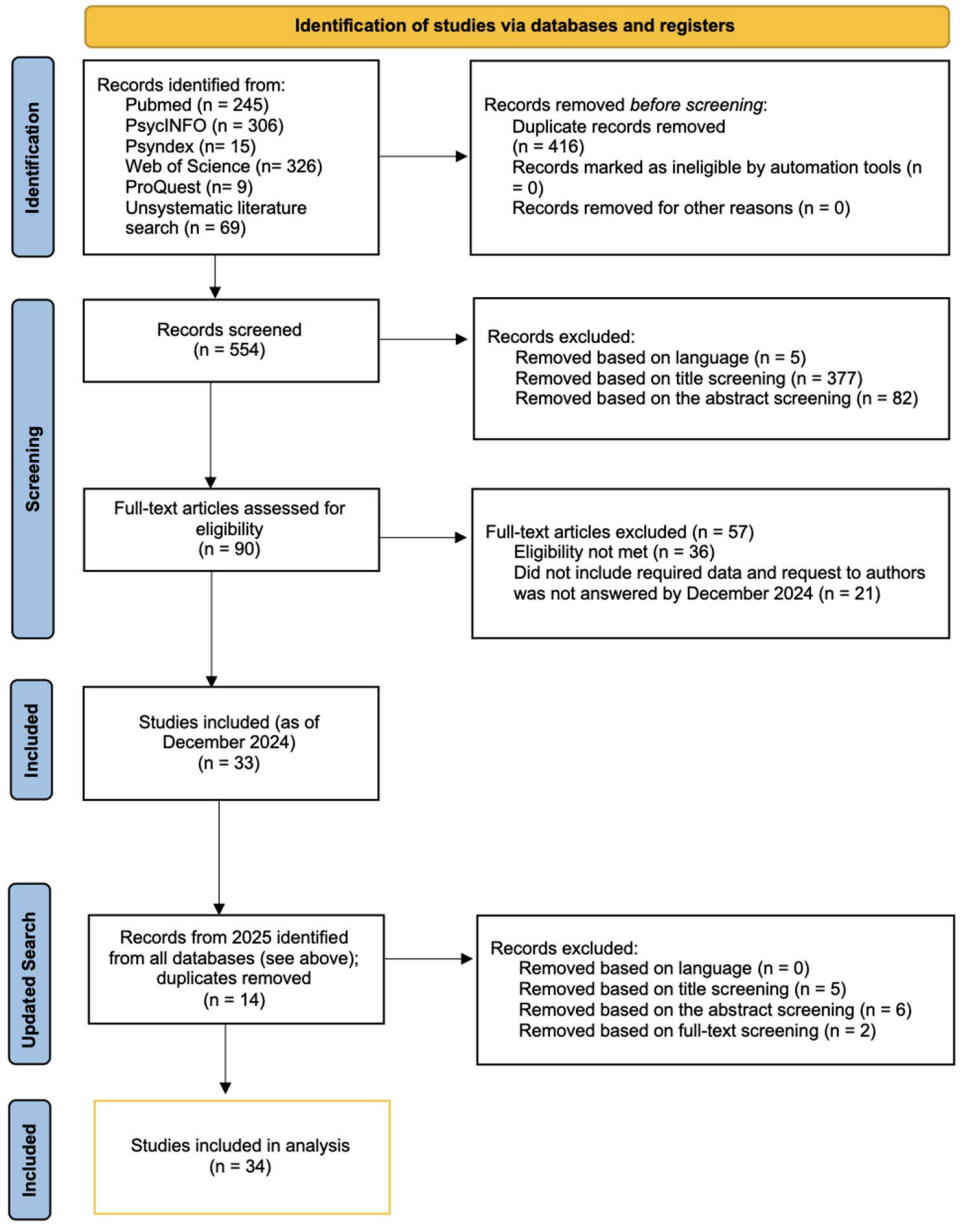
PRISMA flow diagram of study selection. (adapted from [50]). *Notes.* Eligibility not met = Full-text articles were excluded due to the absence of validated measures of BDD symptoms or perfectionism, or because the association between these constructs could not be derived from the reported data

The study selection process is depicted in Fig. 1 (PRISMA flow diagram; [50]). In total, *k* = 34 studies, encompassing 13 107 participants, were included in the final dataset. Additional data were obtained from study authors when necessary.

Included studies were published between 2002 and 2025 and originated from diverse regions, including North America (e.g., Hildebrandt et al. [32], Lividini et al. [45]), Europe (e.g., Borroni et al. [9], Gupta et al. [25]), Asia (e.g., Khanjani et al. [37], Merhy et al. [47], Sadighpour et al. [58]), and Australia/New Zealand (e.g., Hanstock and O’Mahony [27]). Participants’ average age was 25 years, and approximately 55% were women. Analyses included 24 adult samples (e.g., Haider et al. [26], Zoletić et al. [72]), three youth samples [15, 39, 57]), and seven mixed-age samples (e.g., Couper et al. [17], Lamanna et al. [41], Lavell et al. [42]). Nineteen studies involved participants meeting full diagnostic or probable BDD criteria (*n* = 7 862) (e.g., Bartsch [7], Cerea et al. [13], Toh [65]), while fifteen examined subclinical BDD or body dissatisfaction (*n* = 5 246) (e.g., Cunningham et al. [18], Foroughi et al. [22], Yanover et al. [71]). Seven studies included both BDD and mentally healthy controls (*n* = 3 937) (e.g., Johnson et al. [35], Schieber et al. [60]). Nine studies focused on MD symptoms (*n* = 2 546) (e.g., Dryer et al. [19], Grugan et al. [23], Rica et al. [55]).

To assess BDD symptom severity, two studies used interview-based data [21, 29], 29 used self-reports (e.g., Arji et al. [5], Cerea et al. [14]), and three studies used a mixed procedure (e.g., Murray et al. [49]). Eighteen studies used the FMPS or MPS (e.g. Johnson et al. [34]; Pourshahbaz et al. [53]) to assess perfectionism. Of these, five reported the *Concern over Mistakes* subscale and four reported *Doubts about Actions*, both indexing perfectionistic concerns, whereas four studies reported the *Personal Standards* subscale, indexing perfectionistic strivings. Notably, the *Almost Perfect Scale* [62], a commonly used unidimensional measure of perfectionism, would have met the inclusion criteria but was not used in any of the eligible studies. Several studies assessed comorbid symptoms: depression (*k* = 10), OCD (*k* = 3), and EDs (*k* = 8). Table 1 summarizes the main study characteristics.

**Table 1:** Study characteristics and effect sizes

Study (author(s), year)	Year	Country	Design	Recruitment	*N*	% Female	% Male	Age M (SD)	BDD Type	BDD measure	Perfectionism measure	Correlation BDD and perf
Arji et al. [5]	2016	IR	Cross-sectional study	Community	351	77	23	-	Subclinical	BDD-YBOCS	MPS	0.22
Bartsch [7]	2007	AUS	Cross-sectional study	Community	619	73	27	-	Probable BDD	DCQ	MPS	0.29
Borroni et al. [9]	2022	IT	Cross-sectional study	Community	494	100	0	32.66 (14.26)	Subclinical	BDD-D	PSPS	0.40
Cerea et al. [13]	2017	IT	Cross-sectional study	Community	615	69	31	30.51 (13.26)	Probable BDD	DCQ	MPS	0.29
Cerea et al. [14]	2022	IT	Cross-sectional study	Combined	122	89	11	39.89 (14.6)	Probable BDD	QDC	EDI	0.37
Chen et al. [15]	2010	TWN	Cross-sectional study	Community	883	49	51	-	Body dissatisfaction	CDS	EDI	0.25
Couper et al. [17]	2021	GBR	Cross-sectional study	Clinical	311	85	15	32 (12.45)	Subclinical	DAS-24	PSPS	0.36
Cunningham et al. [18]	2018	AUS	Cross-sectional study	Community	106	0	100	21.94 (7.73)	Subclinical	BICI	MPS	0.26
Dryer et al. [19]	2016	AUS	Cross-sectional study	Community	158	0	100	26.94 (5.5)	Subclinical	MDQ	MPS	0.25
Fang et al. [21]	2020	US	Clinical trial	Clinical	45	60	40	35 (10.1)	Established diagnosis	BDD-YBOCS	MPS	0.25
Foroughi et al. [22]	2023	IR	Correlation study	Community	210	51	49	39.2 (10.22)	Subclinical	DCQ	APS	0.25
Grugan et al. [24]	2023	GBR	Correlation study	Community	343	0	100	-	Probable BDD	MDDI	PSPS	0.44
Gupta et al. [25]	2023	GBR	Cross-sectional study	Community	208	64	36	-	Probable BDD	BIQ-C	CAPS	0.36
Haider et al. [26]	2024	CN	Cross-sectional study	Community	270	73	27	19.61 (0.933)	Probable BDD	EDI-BD	FMPS	0.29
Hanstock et al. [27]	2002	AUS	Cross-sectional study	Community	165	100	0	20.2 (2.3)	Subclinical	DCQ	MPS	0.58
Hartmann et al. [30]	2014	DEU	Clinical trial	Combined	69	78	22	28.2 (11.05)	Established diagnosis	BDD-YBOCS	FMPS	0.12
Hildebrandt et al. [32]	2012	US	Clinical trial	Community	174	50	50	19.17 (1.99)	Probable BDD	MDDI	FMPS	0.26
Johnson et al. [34]	2019	AUS	Case series	Community	31	90	10	22.06 (5.54)	Subclinical	DCQ	FMPS	0.23
Johnson et al. [35]	2020	AUS	Cross-sectional study	Community	57	91	9	22.35 (7.24)	Probable BDD	DCQ	FMPS	0.38
Khanjani et al. [37]	2019	IRN	Cross-sectional study	Community	210	51	49	22.1 (2.39)	Subclinical	DCQ	APS	0.22
Krebs et al. [39]	2019	GBR	Longitudinal observational study	Community	302	47	52	-	Probable BDD	BIQ-C	CAPS	0.38
Lamanna et al. [41]	2010	US	Cross-sectional study	Community	199	53	47	19.13 (2.01)	Body dissatisfaction	BSQ	MPS	0.29
Lavell et al. [42]	2014	AUS	Correlation study	Community	246	74	26	21.13 (4.86)	Subclinical	AAI	OBQ-44	0.37
Lividini [45]	2013	US	Cross-sectional study	Community	67	0	100	-	Probable BDD	MASS	MPS	0.46
Merhy et al. [47]	2023	LBN	Cross-sectional study	Community	396	0	100	25.39 (4.96)	Probable BDD	MDDI	BTPS	0.31
Murray et al. [49]	2012	AUS	Clinical trial	Community	119	0	100	21.86 (2.49)	Mixed	MDDI	MPS	0.47
Pourshahbaz et al. [53]	2014	IRN	Correlation study	Community	240	0	100	25.25 (6.39)	Probable BDD	MDDI	MPS	0.31
Rica et al. [55]	2023	ESP	Cross-sectional study	Community	850	0	100	19.9 (2.7)	Probable BDD	MDDI	PAPS	0.34
Rosewall et al. [57]	2018	NZL	Cross-sectional study	Community	207	100	0	15.5 (1.05)	Body dissatisfaction	EDI-BD	CAPS	0.20
Sadighpour et al. [58]	2019	IRN	Correlation study	Community	802	62	38	20.79 (2.1)	Probable BDD	BDD-YBOCS	MPS	0.35
Schieber et al. [60]	2013	DEU	Cross-sectional study	Community	2129	54	46	45.23 (13.1)	Probable BDD	DCQ	EDI	0.24
Toh et al. [65]	2025	AUS	Cross-sectional study	Community	434	76	24	31.5 (10.6)	Probable BDD	DCQ	FMPS	0.45
Yanover et al. [71]	2008	US	Clinical trial	Community	1584	75	25	20.43 (3.71)	Body dissatisfaction	EDI-BD	EDI	0.09
Zoletic et al. [72]	2009	BA	Correlation study	Community	91	100	0	-	Body dissatisfaction	BMI-SMT	NPQ	0.60

*Notes. N* = sample size; % Female and % Male = gender distribution; *M* = mean; *SD* = standard deviation. *BDD type* = diagnostic status (subclinical, probable, or established diagnosis). *Main BDD/perfectionism measure* = primary assessment of BDD symptoms/perfectionism. Measures of BDD symptoms: AAI = Appearance Anxiety Inventory; BDD-D = Body Dysmorphic Disorder–Dimensional Scale; BDD-YBOCS = Yale–Brown Obsessive Compulsive Scale modified for BDD; BICI = Body Image Concern Inventory; BMI-SMT = Body Mass Index Silhouette Matching Test; BSQ = Body Shape Questionnaire; CDS = Contour Drawing Rating Scale; DCQ = Dysmorphic Concern Questionnaire; EDI-BD = Eating Disorder Inventory–Body Dissatisfaction; MASS = Muscle Appearance Satisfaction Scale; MDDI = Muscle Dysmorphic Disorder Inventory; MDQ = Muscle Dysmorphia Questionnaire; QDC = Questionario sul Dismorfismo Corporeo. Measures of perfectionism: APS = Ahvaz Perfectionism Scale; BTPS = Big Three Perfectionism Scale; CAPS = Child–Adolescent Perfectionism Scale; EDI = Eating Disorder Inventory–Perfectionism Subscale; FMPS = Frost Multidimensional Perfectionism Scale; MPS = Multidimensional Perfectionism Scale; NPQ = Neurotic Perfectionism Questionnaire; OBQ-44 = Obsessive Beliefs Questionnaire–Perfectionism and Intolerance of Uncertainty Subscale; PAPS = Physical Appearance Perfectionism Scale; PSPS = Perfectionistic Self-Presentation Scale; although the OBQ-44 is typically used to assess OCD-related beliefs, in one study it was used to operationalize perfectionism via the *perfectionism and intolerance of uncertainty* subscale. *r* = zero-order Pearson correlation between BDD symptom severity and perfectionism. Country codes: AUS = Australia; BA = Bosnia and Herzegovina; CN = China; DEU = Germany; ESP = Spain; GBR = United Kingdom; IR/IRN = Iran; IT = Italy; LBN = Lebanon; NZL = New Zealand; TWN = Taiwan; US = United States

### BDD symptom severity and perfectionism

**Fig. 2:**
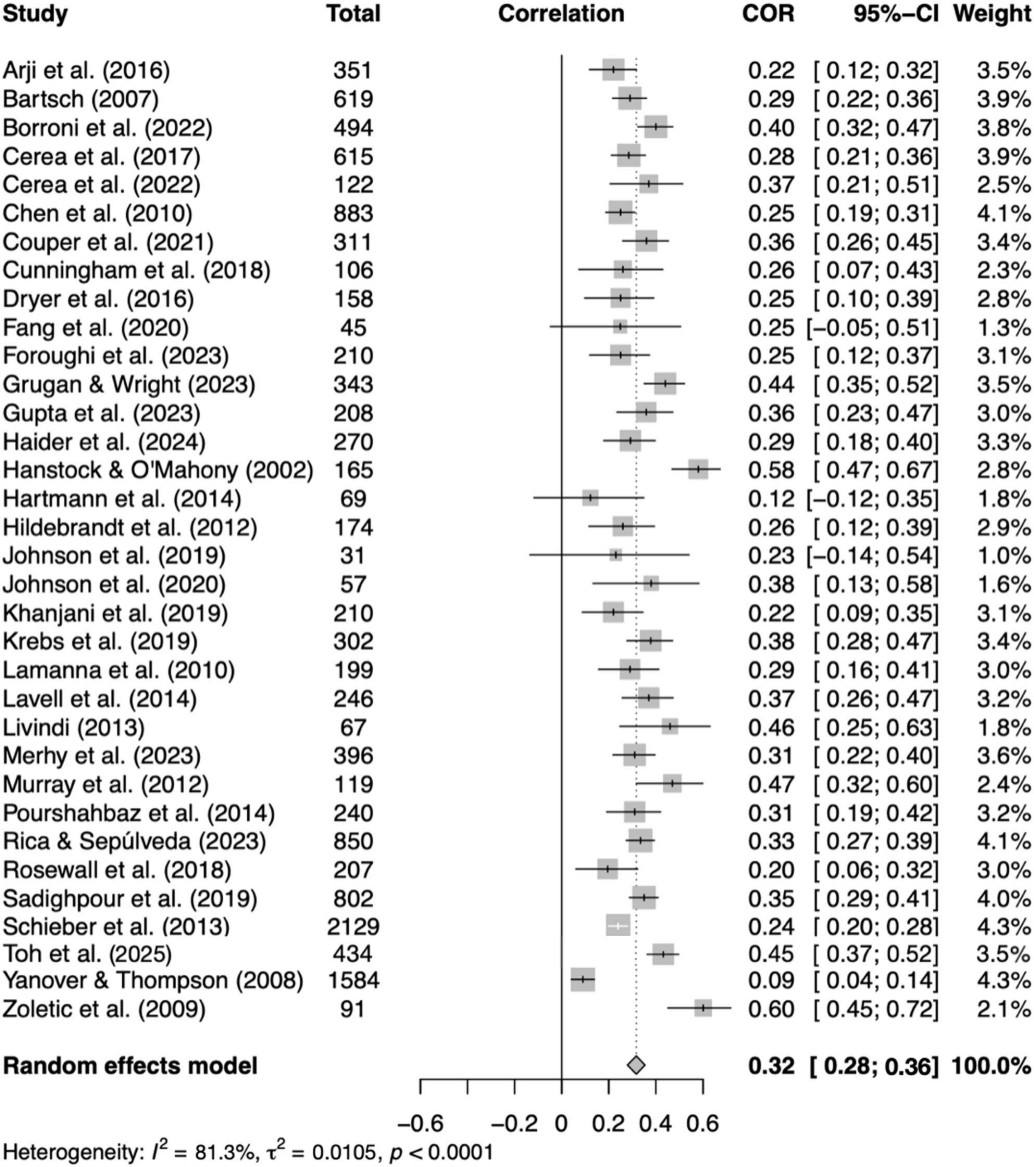
Correlations between BDD symptom severity and perfectionism. *Notes.* Squares represent individual study correlations (r) with 95% confidence intervals; the diamond indicates the pooled random-effects estimate

A random-effects model meta-analysis was performed to examine the zero-order correlations between the severity of BDD symptoms and perfectionism, yielding an overall effect size of *r* = .32, 95% *CI* [0.28, 0.36], *p* < .001, indicating a moderate correlation based on *k* = 34 with a total *N* = 13 107 (see Fig. 2). The analysis revealed considerable heterogeneity across studies (*I²* = 81%) and a small between-study variance (τ² ≈ 0.01), indicating that while effect sizes varied substantially relative to sampling error, the absolute magnitude of between-study variance was modest.

### BDD symptom severity and perfectionism dimensions

**Fig. 3:**
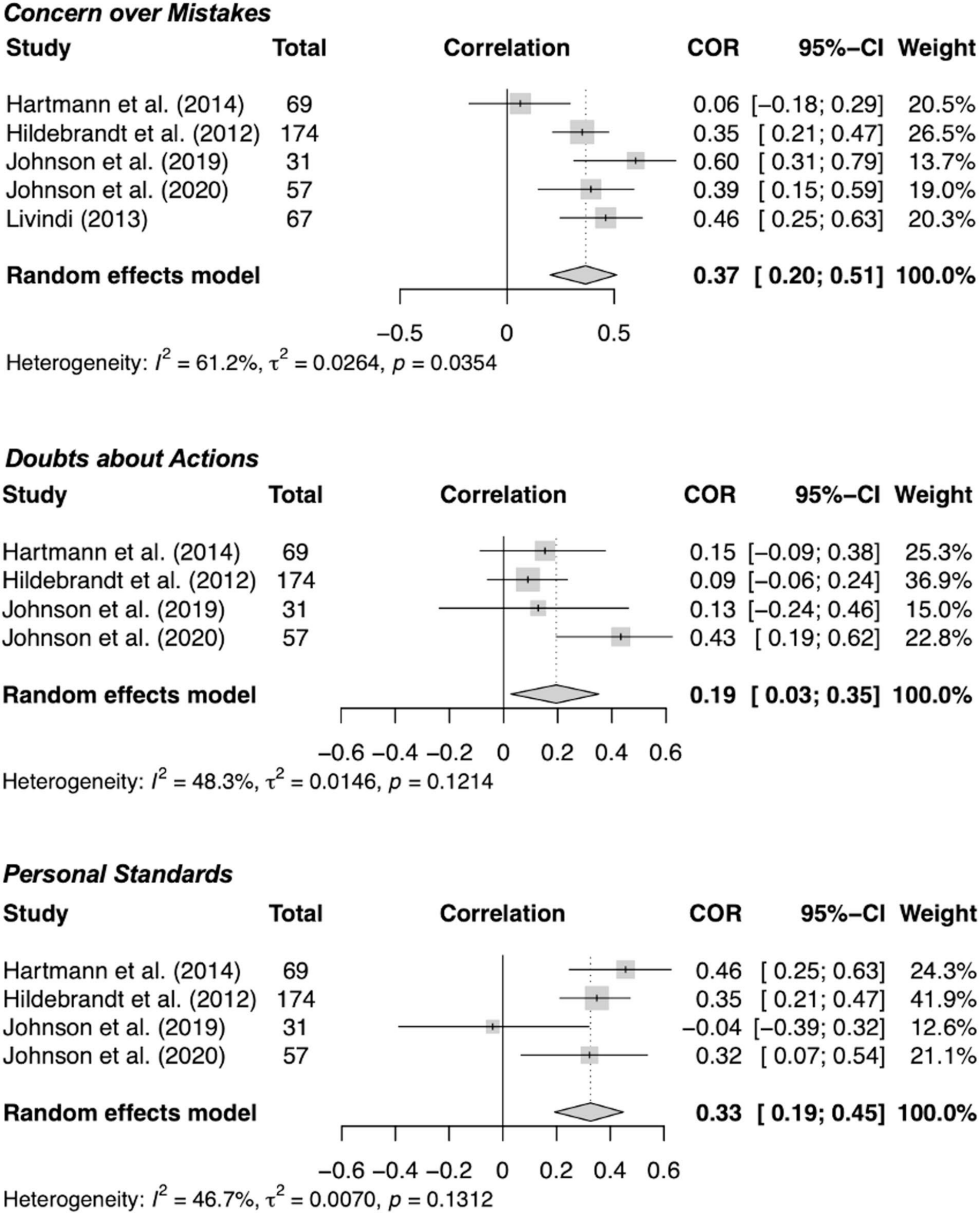
Specific dimensions of perfectionism. *Notes.* Forest plots show correlations between BDD symptom severity and perfectionism subdimensions (Concern over Mistakes, Doubts about Actions, Personal Standards); diamonds represent pooled random-effects estimates

Dimension-specific analyses using available FMPS-based subscales indicated significant positive associations between BDD symptom severity and both concern-related and striving-related facets of perfectionism (see Fig. 3). Significant positive associations emerged for the concern-related subscales *Concern over Mistakes* (*r* = .37, *p* < .001), *Doubts about Actions* (*r* = .19, *p* = .023), as well as for the striving-related subscale *Personal Standards* (*r* = .33, *p* < .001). Heterogeneity within each subscale model was moderate (*I²* = 47–61%), reflecting some variation among the included studies.

### Subgroup differences in the relationship between BDD symptom severity and perfectionism

Subgroup analyses showed no significant between-group differences for age, gender, diagnostic subtype, or clinical status (all *p* > .05). Thus, the magnitude of the association between BDD symptom severity and perfectionism did not differ significantly across these study characteristics. Table 2 presents pooled effect sizes within each subgroup for descriptive comparison, whereas the formal subgroup model outputs are reported in the supplementary material (Figure S1-S4). Specifically, no significant differences were observed between age groups, indicating comparable associations across adult, youth, and mixed-age samples. Similarly, gender did not significantly moderate the association, with moderate effect sizes observed in both male and female samples. No significant differences were found between studies assessing general BDD symptoms and those focusing on MD, suggesting comparable associations across diagnostic subtypes. Likewise, analyses comparing samples meeting full diagnostic or probable BDD criteria (type 1) and those with subclinical symptoms or elevated body dissatisfaction (type 2) showed no significant differences, despite numerically higher effect sizes in clinically defined samples.

**Table 2:** Descriptive summary of subgroup-specific pooled effect sizes

Moderator	Subgroup	k	*r*	95% CI	*p*
Age	Adult	24	0.32	[0.27, 0.37]	< 0.001
	Youth	3	0.28	[0.18, 0.37]	< 0.001
	Mixed-age	7	0.32	[0.28, 0.35]	< 0.001
Gender	Male	8	0.35	[0.24, 0.45]	< 0.001
	Female	4	0.45	[0.26, 0.61]	< 0.001
Diagnostic subtype	BDD symptoms	24	0.31	[0.26, 0.36]	< 0.001
	MD symptoms	9	0.34	[0.29, 0.39]	< 0.001
Clinical status	BDD type 1	19	0.32	[0.29, 0.36]	< 0.001
	BDD type 2	15	0.31	[0.23, 0.38]	< 0.001

*Notes. k* = number of studies; *r* = pooled zero-order Pearson correlation. BDD type 1 = studies including participants meeting full/probable diagnostic criteria; BDD type 2 = studies with subclinical/elevated body dysmorphic concerns. For the gender analysis, only studies with exclusively female or male samples were included and for the diagnostic subtype analysis, only studies assessing exclusively BDD or MD symptoms were included

### Association between BDD symptom severity and perfectionism when controlling for depression

A separate random-effects meta-analysis of partial correlations controlling for depressive symptoms showed that the association between BDD symptom severity and perfectionism remained positive but was substantially attenuated relative to the zero-order effect (*r* = .09, 95% CI [0.02, 0.16], *p* = .011). Only *k* = 5 studies provided sufficient intercorrelation data among BDD symptom severity, perfectionism, and depression to permit computation of partial correlations and inclusion in this analysis. Heterogeneity was low to moderate (*I*² = 24.8%). Individual study-level estimates varied (*r* = − .06 to 0.15) but should be interpreted cautiously given the small number of included studies.

### Sensitivity analysis and publication bias

Of the 34 studies, three were rated as low risk of bias, 14 moderate, and 17 high. Risk of bias did not significantly moderate the association (*p* ≥ .05). Leave-one-out analyses confirmed the stability of the overall effect; even after excluding two large-scale studies [60, 71], the pooled correlation decreased slightly but remained within the same magnitude and direction as the main estimate. Funnel plot inspection revealed asymmetry, with smaller studies tending to report larger effects (supplement Figure S5). Egger’s regression confirmed significant small-study effects (bias estimate = 2.08, *SE* = 0.76, *p* = .010), suggesting possible publication bias. Subgroup analysis by sample type (student, community, clinical, mixed) yielded largely consistent results, supporting the consistency and generalizability of the association. The corresponding forest plot is provided in the supplement (Figure S6).

### Meta-analysis of selective studies: BDD symptom severity and perfectionism in individuals with body image-related distress

**Fig. 4:**
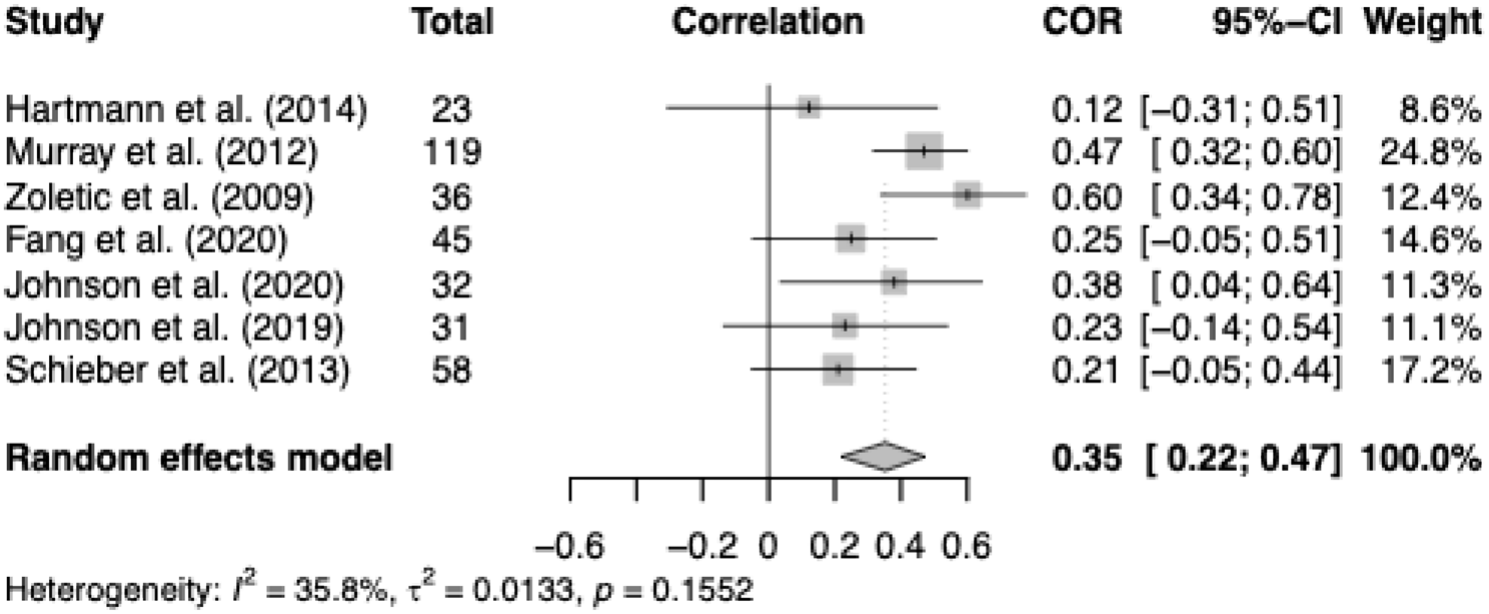
Correlations between BDD symptom severity and perfectionism in secondary meta-analysis. *Notes.* Squares indicate study-specific correlations (r) with 95% confidence intervals; the diamond shows the pooled random-effects estimate based on studies with elevated BDD symptoms

In a subset of seven studies including only participants with elevated body image-related distress (*n* = 344 adults with a mean age of 28 years; 68% female), the random-effects model indicated a moderate positive correlation (*r* = .35, 95% CI [0.22, 0.47], *p* < .001), with moderate heterogeneity (*I²* = 35.8%, see Fig. 4). Two samples included clinically diagnosed BDD participants (*n* = 68), three met probable full diagnostic criteria (*n* = 209), and two showed subclinical BDD symptoms or body dissatisfaction (*n* = 67). In one sample, MD symptoms were assessed as a related manifestation of BDD. Three studies used the FMPS or MPS as the main measure of perfectionism. Fewer than three studies assessed depressive, OCD, or ED symptoms, precluding the inclusion of these variables in the meta-analysis of partial correlations.

Five studies were rated as moderate risk of bias and two as high. Bias did not significantly moderate the association between BDD symptom severity and perfectionism (*p* ≥ .05). Funnel plot inspection showed no extreme outliers and relatively narrow confidence intervals, consistent with the moderate heterogeneity (supplement Figure S7). Sensitivity analysis excluding subclinical samples [34, 72] yielded a comparable effect size (*r* = .33, 95% CI [0.19, 0.46]), with lower but still moderate heterogeneity (*I²* = 26.5%), confirming the consistency of the association.

## Discussion

This meta-analysis examined the cross-sectional relationship between BDD symptom severity and perfectionism across 34 studies encompassing over 13 000 participants. Overall, a moderate positive zero-order correlation emerged, indicating that higher BDD symptom severity co-occurs with elevated levels of perfectionism. Specifically, these associations were observed for self-critical and evaluative facets of perfectionistic concerns, as well as for elevated personal standards reflecting perfectionistic strivings. This pattern is compatible with cognitive-behavioral accounts of BDD, which emphasize rigid self-evaluation, intolerance of perceived imperfection, and heightened appearance-related standards as central features of symptom expression and distress [66, 69]. From this perspective, perfectionistic tendencies may reflect heightened appearance-based self-scrutiny and evaluative threat, co-occurring with checking, comparison, and corrective behaviors characteristic of BDD. At the same time, the cross-sectional nature of the evidence precludes conclusions regarding directionality, such that the observed association may reflect perfectionism as a vulnerability-related feature, a correlational aspect of symptom maintenance, or broader overlap with core BDD-related cognitions, rather than indicating causal precedence.

Extending these findings, the association remained comparable in a secondary analysis restricted to individuals with elevated BDD symptoms, indicating that perfectionism is consistently associated with symptom expression even in samples meeting full diagnostic criteria and/or receiving treatment. This finding underscores the clinical relevance of perfectionism in BDD-related distress and aligns with theoretical accounts emphasizing its co-occurrence with appearance-focused psychopathology [9].

Across subgroups, analyses revealed stable effects across age groups and sample types. While numerically stronger in female-only samples, gender differences were non-significant, indicating that perfectionism is associated with BDD symptoms irrespective of demographic characteristics, with gender-related differences likely reflecting variation in the magnitude rather than the nature of the association. Similarly, associations were comparable across BDD and MD, suggesting that perfectionism represents a shared correlational feature across appearance-related distress, although its specific content may vary as a function of gender and sociocultural context [19, 24]. It is possible that existing perfectionism measures do not adequately capture muscle-oriented perfectionism, which may be particularly relevant in MD [11].

In line with these findings, comparable patterns have been observed in related disorders such as EDs, where perfectionistic concerns are (similarly to BDD) linked to appearance-based self-evaluation, with the cognitive focus of these concerns differing by gender. For instance, in men, such concerns often center around muscularity, physical strength, and the desire for control and discipline [11, 55], whereas in women, thinness, low body weight, and flawlessness are more frequently idealized [9, 33, 57].

When considering the strength of these associations, interpretation should account for the methodological quality of the underlying evidence base. A substantial proportion of included studies were rated as having moderate to high risk of bias, primarily due to convenience sampling, exclusive reliance on self-report measures, and cross-sectional designs. These features may systematically inflate observed associations between perfectionism and BDD symptom severity through shared method variance, response styles, and overlap in negatively valanced item content. Together with the observed small-study effects, this suggests that the pooled estimate may reflect an upper-bound estimate and could correspond to a smaller effect in methodologically rigorous studies [8].

Regarding depressive symptoms, the pooled partial correlation was small but remained statistically significant, indicating that the association between BDD symptom severity and perfectionism is substantially attenuated when accounting for depression. This suggests that part of the zero-order association may reflect broader negative affectivity and shared features such as self-critical evaluation and sensitivity to perceived failure [33, 65]. Conceptual overlap between constructs, as well as shared method variance (e.g., negatively valenced item content), may further contribute to inflated unadjusted associations. Accordingly, these findings are consistent with the interpretation of perfectionism as a transdiagnostic correlate rather than a BDD-specific factor [20]. However, because only five studies provided sufficient data, conclusions regarding specificity remain preliminary. Due to limited data, comparable analyses for OCD or ED symptoms were not feasible in the present meta-analysis, although existing theoretical and empirical work [12, 59] suggests that these conditions may also influence observed correlations.

Dimension-specific analyses indicated positive associations between BDD symptom severity and both concern-related and striving-related facets of perfectionism. *Concern over Mistakes* (concern-related) and *Personal Standards* (striving-related) showed the strongest associations, followed by *Doubts about Actions* (concern-related). Rather than reflecting an unitary construct, these findings point to heterogeneity across perfectionism dimensions [61]. Self-critical and evaluative facets may be especially relevant in the context of appearance-related distress, given their conceptual overlap with heightened self-scrutiny and intolerance of perceived imperfection emphasized in cognitive-behavioral models of BDD [67]. In contrast, the striving-related association may reflect rigid appearance-related ideals that co-occur with symptom severity rather than processes directly tied to uncertainty or checking. Together, these findings suggest that distinguishing between higher-order perfectionistic concerns and strivings may be informative, as reliance on global perfectionism scores could obscure theoretically meaningful heterogeneity in their associations with BDD symptom severity [61].

In sum, this meta-analysis provides quantitative evidence for a moderate and consistent association between perfectionism and BDD symptom severity, with stable associations observed for both concern-related (self-critical) and striving-related (personal standards) dimensions of perfectionism. By synthesizing effect sizes across studies and examining dimension-specific patterns, the present findings extend previous reviews on related disorders and constructs [46, 52] and clarify both the overall strength and the higher-order dimensional specificity of the association between perfectionism and BDD symptom severity.

### Limitations

Several limitations should be considered when interpreting the present findings. First, conclusions are constrained by the exclusively cross-sectional nature of the included studies. Consequently, causal inferences regarding the temporal relationship between perfectionism and BDD symptom severity cannot be drawn. It remains unclear whether perfectionism represents a correlational feature, an epiphenomenon, or a consequence of BDD, highlighting the need for longitudinal designs.

Dimensional analyses of perfectionism were based on subscale-level data available in only a subset of studies, precluding a comprehensive synthesis of higher-order perfectionism constructs across instruments. As a result, facet-level estimates relied primarily on FMPS-based subscales and should be interpreted as providing an indicative illustration of the distinction between perfectionistic concerns and strivings, rather than as definitive estimates of higher-order perfectionism dimensions. This underscores the need for more consistent reporting of theoretically grounded perfectionism dimensions in future research. Relatedly, one included study [42] operationalized perfectionism using a subscale not originally developed as a trait perfectionism measure (the OBQ-44 subscale *perfectionism and intolerance of uncertainty*), underscoring the need for more standardized and theoretically grounded assessment of perfectionism in future BDD research.

A further limitation concerns the moderator analyses, which were constrained by inconsistent reporting of diagnostic methods, comorbid psychopathology, and other variables. Gender representation was also uneven, with male samples frequently focused on MD [24, 55] and female samples on broader BDD [9, 57], reducing comparability. Moreover, gender-diverse individuals were largely absent from the literature, limiting generalizability despite evidence of elevated body-related distress in gender-diverse populations [1].

Few studies employed clinician-administered diagnostic interviews, and comorbidity data were too inconsistently reported to allow systematic meta-analytic adjustment beyond depression. Consequently, comparable analyses controlling for OCD or ED symptoms were not feasible in the present meta-analysis, despite theoretical and empirical evidence suggesting that these conditions may also influence the observed associations [52, 59]. Furthermore, only a small number of studies reported sufficient intercorrelation data to compute partial correlations controlling for depressive symptoms. Given evidence that partial correlation estimates can be unstable in smaller samples [63], these adjusted findings should be interpreted cautiously. This limitation also constrains conclusions regarding the specificity of the association between perfectionism and BDD when controlling for depressive symptoms.

Substantial heterogeneity was observed across studies, likely reflecting wide variation in BDD symptom severity and differences in assessment methods. Consistent with this interpretation, heterogeneity was reduced in the secondary meta-analysis restricted to samples with elevated BDD symptoms. Although random-effects models were applied, this variability limits the comparability of effect sizes, particularly given the use of heterogeneous instruments ranging from structured clinical interviews [21, 30] to brief self-report screening measures [27, 49], which may lack diagnostic specificity and blur distinctions between BDD-specific concerns and symptoms related to other disorders [36, 48].

In addition, evidence of publication bias and small-study effects was detected, with smaller studies tending to report larger associations. Together with the presence of small samples, this pattern suggests that the pooled effect size may represent an upper-bound estimate of the true association [8]. Although sensitivity analyses indicated overall robustness and risk of bias did not significantly moderate effect sizes, the absence of moderation does not preclude systematic overestimation and therefore warrants cautious interpretation.

Finally, despite its theoretical relevance to BDD [41, 55], appearance-based perfectionism could not be examined due to insufficient standardized data, highlighting an important gap in the existing literature. Thus, the observed effect should be interpreted cautiously and may be closer to a small effect under more rigorous conditions.

### Clinical implications

The present findings have implications for the assessment and clinical conceptualization of BDD and related appearance-focused disorders. Across samples, perfectionism (particularly self-critical and evaluative dimensions) was consistently associated with BDD symptom severity, suggesting that these characteristics may be clinically informative correlates of symptom expression. Incorporating assessment of perfectionistic concerns may therefore aid case formulation and hypothesis generation in clinical settings [61].

Although intervention effects were not evaluated in this meta-analysis, the consistent associations observed for both concern-related and striving-related dimensions of perfectionism highlight facets that warrant further investigation in treatment-oriented research (e.g., error intolerance and inflexible standards). Established cognitive-behavioral strategies such as cognitive restructuring, behavioral experiments, and exposure [28] could be considered in future work examining whether cognitive-behavioral techniques that address perfectionistic beliefs are relevant for BDD presentations.

Associations were also evident in samples with elevated BDD symptoms, indicating that perfectionism remains associated with symptom severity even at higher levels, underscoring its relevance for clinical conceptualization [34]. In addition, the comparable pattern of associations observed for BDD and MD suggests that perfectionism represents a shared correlational feature across appearance-related distress, albeit with content-specific manifestations (e.g., thinness versus muscularity ideals) [19]. These considerations may be relevant for tailoring clinical hypotheses pending treatment-efficacy evidence.

Moreover, early identification of perfectionistic thinking may be relevant for understanding correlates of elevated BDD symptoms in early stages. Youth facing high sociocultural appearance pressures may be an important target group for future research evaluating interventions that address perfectionism and self-compassion in relation to BDD symptoms [2]. Gender-sensitive approaches are essential, especially in MD, where muscularity standards and appearance-contingent self-worth play a central role [19].

Overall, our findings highlight perfectionism as a clinically relevant correlate of BDD symptom severity. Its causal and therapeutic relevance should be tested in longitudinal, experimental, and intervention studies.

### Future directions

Future research should clarify the directional relationship between BDD symptom severity and perfectionism. While our findings are compatible with theoretical models in which perfectionism is relevant to BDD symptom processes, longitudinal and experimental research is needed to test temporal ordering and potential causal pathways [57, 60].

Further work should also identify subgroups in which perfectionistic tendencies are especially pronounced and examine moderators such as gender identity, cultural background, and personality traits. Sociocultural influences, including media portrayals, may differentially shape muscularity-oriented perfectionism in MD and flawlessness-oriented perfectionism in other forms of BDD, reflecting gender norms [11, 33].

Distinguishing perfectionistic concerns from striving-related dimensions of perfectionism is another priority. Evidence indicates that perfectionistic concerns, such as *Concern over Mistakes*, appear most consistently associated with BDD symptoms [26, 30], whereas more achievement-oriented or self-enhancing aspects of perfectionism may be related to symptom expression through different or more indirect pathways, for instance via narcissistic traits [26]. Clarifying these distinctions could improve theoretical models and inform risk identification for more persistent or treatment-resistant symptom trajectories [20].

Future research would benefit from consistent use and reporting of well-validated perfectionism measures and theoretically grounded subdimensions. Improved measurement consistency would facilitate synthesis across studies and allow more precise examination of appearance-specific perfectionism. Especially in populations in which appearance is central (e.g., athletes, cosmetic surgery patients), perfectionism and BDD may manifest differently and require tailored assessment approaches [14, 19].

Experimental and intervention research is needed to evaluate whether interventions targeting perfectionism are associated with reductions in BDD symptom severity [61]. Research in the context of depressive disorders has shown that beliefs centered around personal inadequacy, characterized by rigid standards and fear of failure, function as a maintaining mechanism [64], raising the possibility that interventions challenging perfectionistic beliefs could be evaluated as a potential adjunct in BDD treatment research, pending empirical evaluation.

Finally, interventions fostering self-compassion warrant investigation. Self-compassion has been linked to more stable self-worth and fewer harmful appearance-based comparisons [44]. Indeed, initial findings indicate an association between higher self-compassion and lower BDD symptoms in adolescents [2], suggesting its potential as a complementary treatment component.

## Conclusion

This meta-analysis provides the first quantitative synthesis of the cross-sectional relationship between BDD symptom severity and perfectionism. Across 34 studies, a moderate positive relationship emerged, with associations observed for both concern-related and striving-related facets of perfectionism. These findings indicate that higher BDD symptom severity co-occurs with greater perfectionistic tendencies, particularly those reflecting rigid standards and harsh self-evaluation. They further support theoretical models emphasizing perfectionistic self-evaluation and rigid standards as relevant processes in BDD, while also suggesting that perfectionism may reflect a broader transdiagnostic correlate rather than a BDD-specific mechanism. Given the consistency of the association across samples and subgroups, assessing perfectionism may be clinically informative in the context of BDD. However, because the available evidence is cross-sectional, causal inferences cannot be drawn. Longitudinal and experimental studies are needed to clarify directionality and to test whether interventions that address perfectionism are associated with changes in BDD symptom severity.

## Supplementary Information

Below is the link to the electronic supplementary material.

Supplementary Material 1

## Data Availability

The complete dataset, analysis script, and all additional materials supporting this meta-analysis have been uploaded to OSF. The full PRISMA 2020 checklist is also available. Link: (https://osf.io/dk7cn/overview?view_only=17b9bdb6cce841e48c04fff97799c5a8) .

## Abbreviations

APA: American Psychiatric Association
BDD: Body Dysmorphic Disorder
DSM: Diagnostic and Statistical Manual of Mental Disorders
ED: Eating Disorder
ICC: Intraclass Correlation Coefficient
ICD: International Classification of Diseases
MD: Muscle Dysmorphia
OCD: Obsessive–Compulsive Disorder
OSF: Open Science Framework
PRISMA: Preferred Reporting Items for Systematic Reviews and Meta-Analyses
REML: Restricted Maximum Likelihood estimator
