# Supplementary material for: The cross-sectional relationship between body dysmorphic disorder and perfectionism: a meta-analysis
Source: BMC Psychiatry. 2026 Apr 21;26:338. doi: 10.1186/s12888-026-08044-7 (PMC13101208; doi:10.1186/s12888-026-08044-7)
Supplement: Supplementary file 1 — Supplementary Material 1 [file 12888_2026_8044_MOESM1_ESM.docx]

Supplementary material

Measure for BDD symptoms:

AAI Appearance Anxiety Inventory

BDD-D Body Dysmorphic Disorder-Dimensional Scale

BDD-YBOCS Yale-Brown Obsessive Compulsive Scale modified for BDD

BICI Body Image Concern Inventory

BIS Body Investment Scale

BMI-SMT Body Mass Index Silhouette Matching Test

BSQ Body Shape Questionnaire

CDS Contour Drawing Rating Scale

DCQ Dysmorphic Concern Questionnaire

EDI-BD Eating Disorders Inventory-Body Dissatisfaction

MASS Muscle Appearance Satisfaction Scale

MDDI Muscle Dysmorphic Disorder Inventory

MDQ Muscle Dysmorphia Questionnaire

QDC Questionario sul Dismorfismo Corporeo

The most used measures for BDD symptoms were the DCQ, MDDI, BDD-YBOCS and the EDI-BD. Further instruments employed in single studies were the AAI, BDD-D, BICI, BIS, BMI-SMT, BSQ, CDS, MASS, MDQ and QDC.

Measure for perfectionism:

APS Ahvaz Perfectionism Scale

BTPS Big Three Perfectionism Scale

CAPS Child‑Adolescent Perfectionism Scale

EDI Eating Disorder Inventory: Perfectionism Subscale

FMPS Frost Multidimensional Perfectionism Scale

MPS Multidimensional Perfectionism Scale

NPQ Neurotic Perfectionism Questionnaire

OBQ-4 Obsessive Beliefs Questionnaire: Perfectionism Subscale

PAPS Physical Appearance Perfectionism Scale

PSPS Perfectionistic Self‑Presentation Scale

The most used measures for perfectionism were the FMPS, MPS, EDI, PSPS, CAPS and the APS. Additional instruments used in individual studies included the BTPS, NPQ, OBQ-4 and PAPS.

Search algorithm

(BDD OR "body dysmorphic concern" OR "body dysmorphic disorder" OR "body dysmorphia" OR "body dysmorphic" OR dysmorphic OR dysmorphia OR dysmorphophobia OR "muscle dysmorphia" OR "dysmorphic concern" OR "dysmorphic concern questionnaire" OR "BDD-YBOCS" OR MDD OR "Body Image disturbance" OR "body image disorder" OR "body dissatisfaction" OR "Muscle dysmorphic disorder inventory") AND (perfectionism OR perfectionist* OR "Frost Multidimensional Perfectionism Scale" OR FMPS OR "PAPS" OR "Perfectionistic Self-Presentation Scale" OR "concern over mistakes" OR "doubts about actions" OR "high personal standards").

Details on the coding procedure

The coding file contained the following information: First, the sample was described regarding the study characteristics including author(s), year of publication, citation, country and study design. Second, the sample characteristics were captured including the sample type (e.g., community, clinical), recruitment method, sample size, age (mean, SD, range) and gender. As well as the characterization of the BDD group (group size, age, gender), diagnosis type (e.g., established diagnosis, reported diagnosis, BDD concern) and potential control group (group size, age, gender). Third was the assessment of constructs including the BDD diagnosis method (e.g., clinical interview, cut-off, self-report) vs. BDD concern (e.g., questionnaire, interview), criteria, instruments used to capture BDD (name, reliability), subgroups, instruments used to capture perfectionism (name, reliability), details on the usage of the FMPS, comorbidities (depression, OCD, ED, other) and comorbidity-details (e.g., diagnosis, mean, SD, diagnosis method, instruments used to capture comorbidity). Subsequently, the relevant outcomes were described, consisting of the correlation between BDD and perfectionism as first outcome and the correlation between perfectionism and other moderating factors as second outcome. In case of absence of reported correlations and the availability of solely subscale data, weighted means were computed to synthesize the information. All variables of interest were required to be assessed at a single time point to ensure a uniform temporal reference. When multiple measurements were available, baseline data was selected for analysis. Additionally, effect size characteristics were investigated.

*
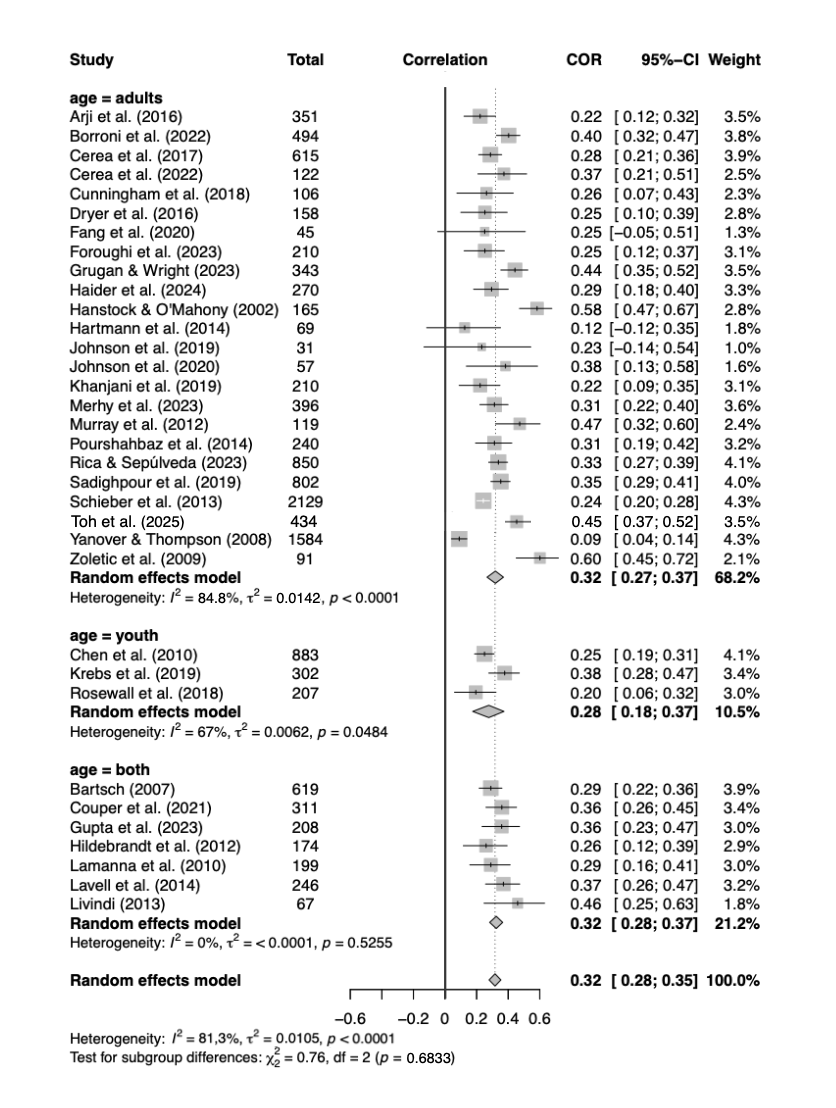
*

*Figure S1.* Forest plot of the association between BDD symptoms and perfectionism by age group.

Note. Subgroup analysis of effect sizes for adults (≥ 18 years), youth (< 18 years), and mixed-age samples (both adults and youth). Squares represent study-specific correlations with 95% CIs, diamonds represent pooled estimates under a random-effects model.

*
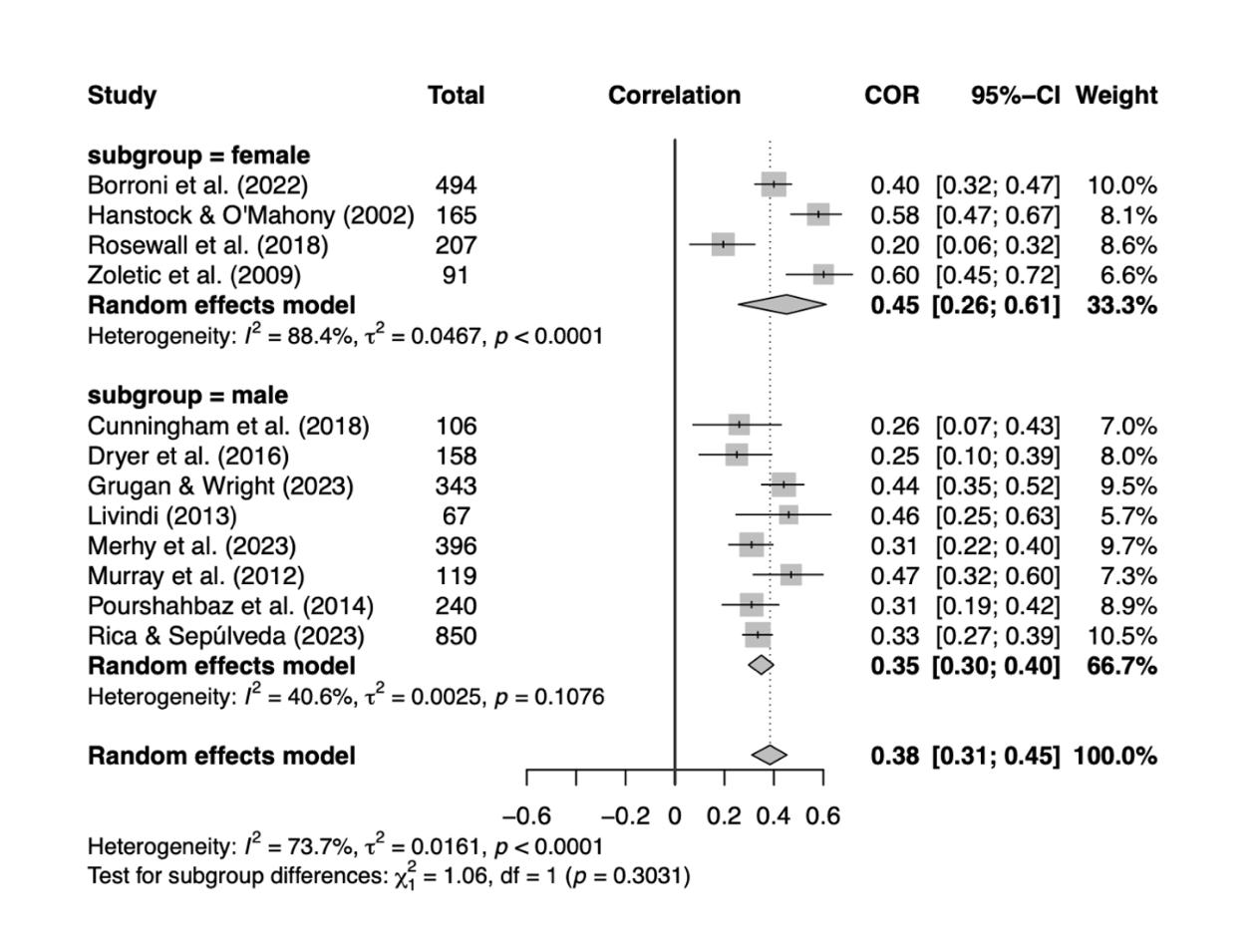
*

*Figure S2.* Forest plot of the association between BDD symptoms and perfectionism by gender group.

Note. Subgroup analysis comparing male samples (0% female participants) and female samples (100% female participants). Squares represent study-specific correlations with 95% CIs, diamonds represent pooled estimates under a random-effects model.


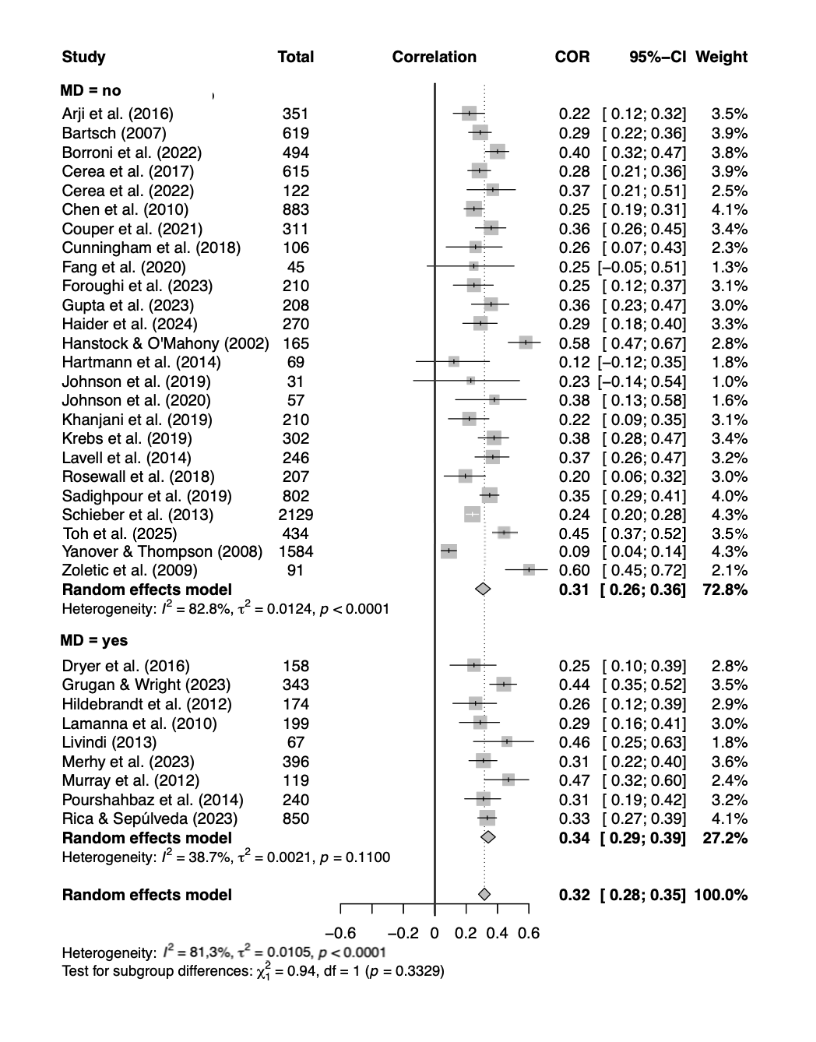


*Figure S3.* Forest plot of the association between BDD symptoms and perfectionism by diagnostic subtype (BDD vs. MD).

Note. Subgroup analysis comparing studies assessing BDD symptoms without muscularity dysmorphia (MD = no) and studies specifically assessing MD symptoms (MD = yes). Squares represent study-specific correlations with 95% CIs, diamonds represent pooled estimates under a random-effects model.

*
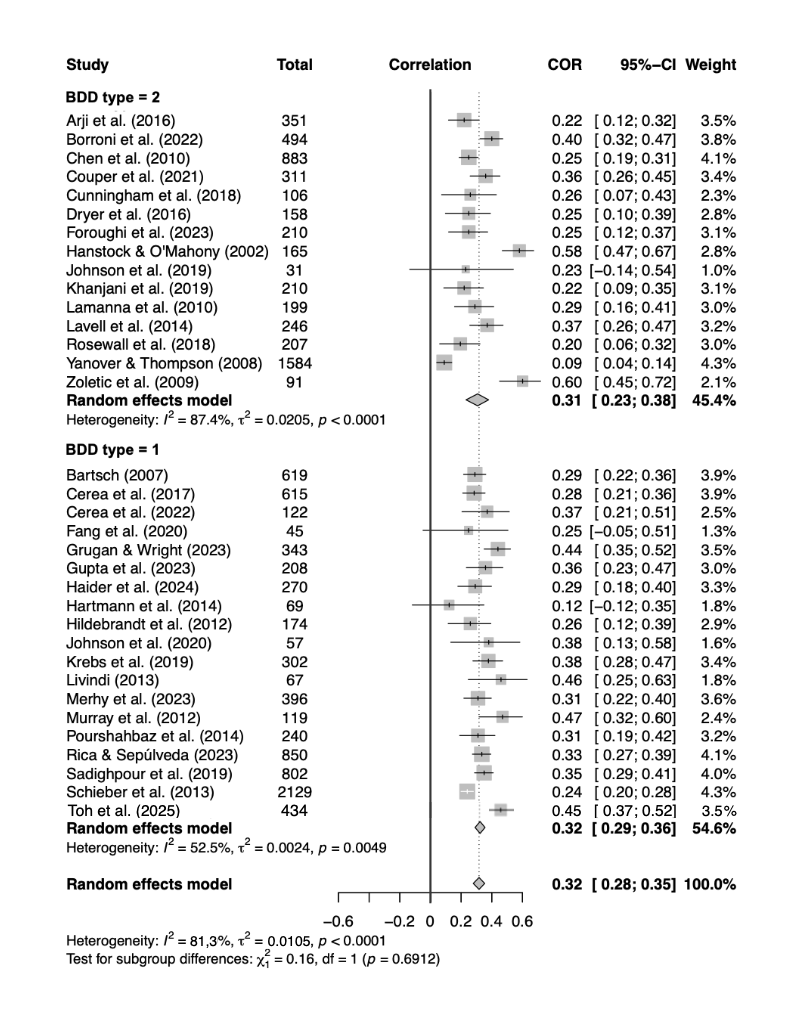
*

*Figure S4.* Forest plot of the association between BDD symptoms and perfectionism by BDD type based on intensity level of expression.

Note. Subgroup analysis comparing clinical/probable BDD (type 1) and subclinical BDD/body dissatisfaction (type 2). Type 1 includes participants with structured expert-rated/self-reported diagnosis, or probable BDD based on established cut-off scores. Type 2 includes participants with elevated BDD concern/body dissatisfaction. Squares represent study-specific correlations with 95% CIs, diamonds represent pooled estimates under a random-effects model.

*Table S1.* Partial Correlations between BDD symptoms and perfectionism controlling for depression.

| Study  (authors, year) | Correlation BDD, perfectionism | Correlation BDD, depression | Correlation perfectionism, depression | Partial correlation BDD, depression, perfectionism |
| --- | --- | --- | --- | --- |
| Cerea et al., 2017 | .285 | .403 | .242 | .146 |
| Cerea et al., 2022 | .37 | .57 | .43 | .067 |
| Gupta et al., 2023 | .359 | .597 | .258 | .004 |
| Hartmann et al., 2014 | .122 | .418 | -.088 | -.064 |
| Krebs et al., 2019 | .378 | .537 | .365 | .126 |

Note. Meta-analysis of partial correlations between BDD symptom severity and perfectionism controlling for depressive symptoms.


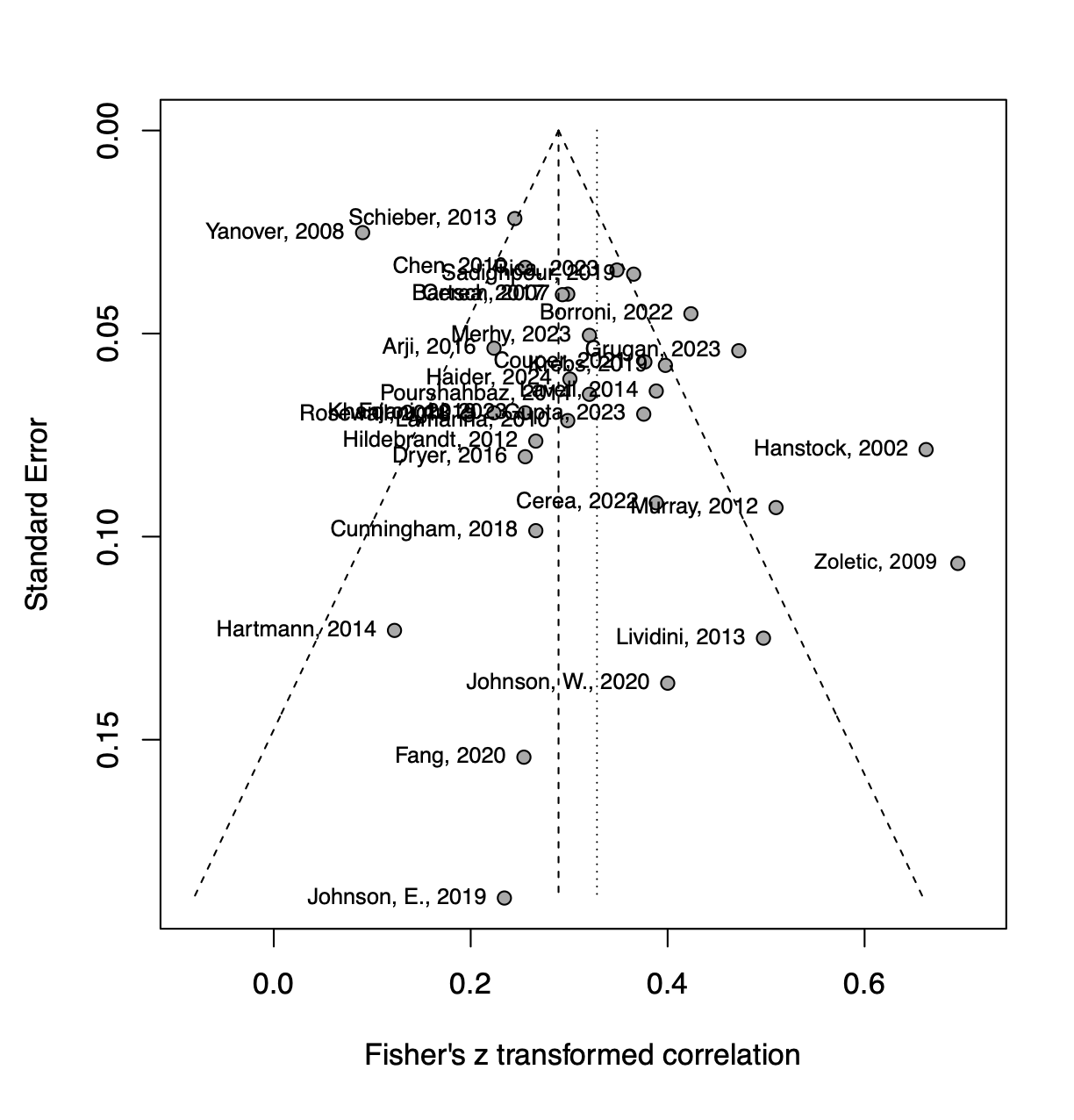


*Figure S5.* Funnel plot of publication bias assessment for primary meta-analysis.

Note. Funnel plot illustrating potential publication bias in the primary meta-analysis of the association between BDD symptom severity and perfectionism. Circles represent individual study effect sizes (Fisher’s z transformed correlations) plotted against their standard errors. The dashed lines indicate the pseudo 95% confidence limits around the pooled effect estimate. To enhance clarity and readability, study labels in the funnel plot were abbreviated to the first author’s last name followed by the publication year (author, year).


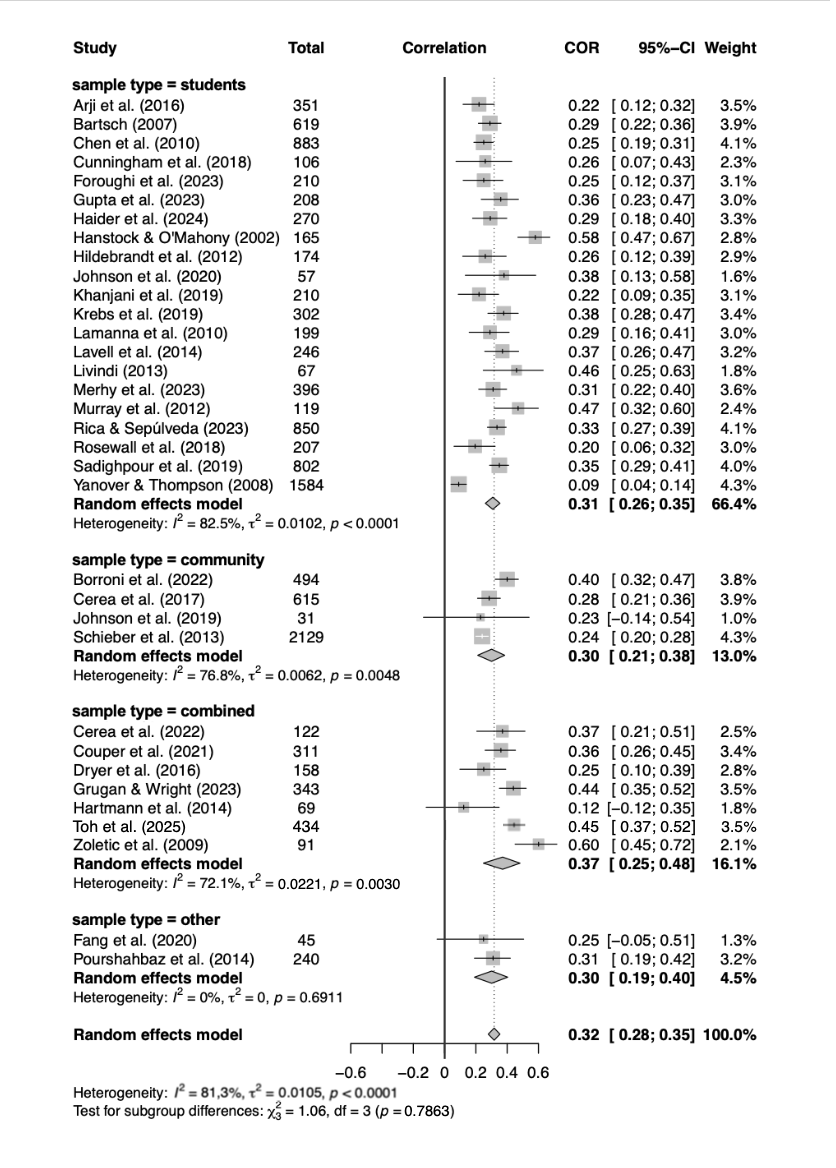


*Figure S6.* Forest plot of the sensitivity analysis by sample type.

Note. Subgroup analysis as part of the sensitivity analysis comparing effect sizes across different sample types: student, community, combined, other (e.g., athlete samples). Squares represent study-specific correlations with 95% CIs, diamonds represent pooled estimates under a random-effects model.

**
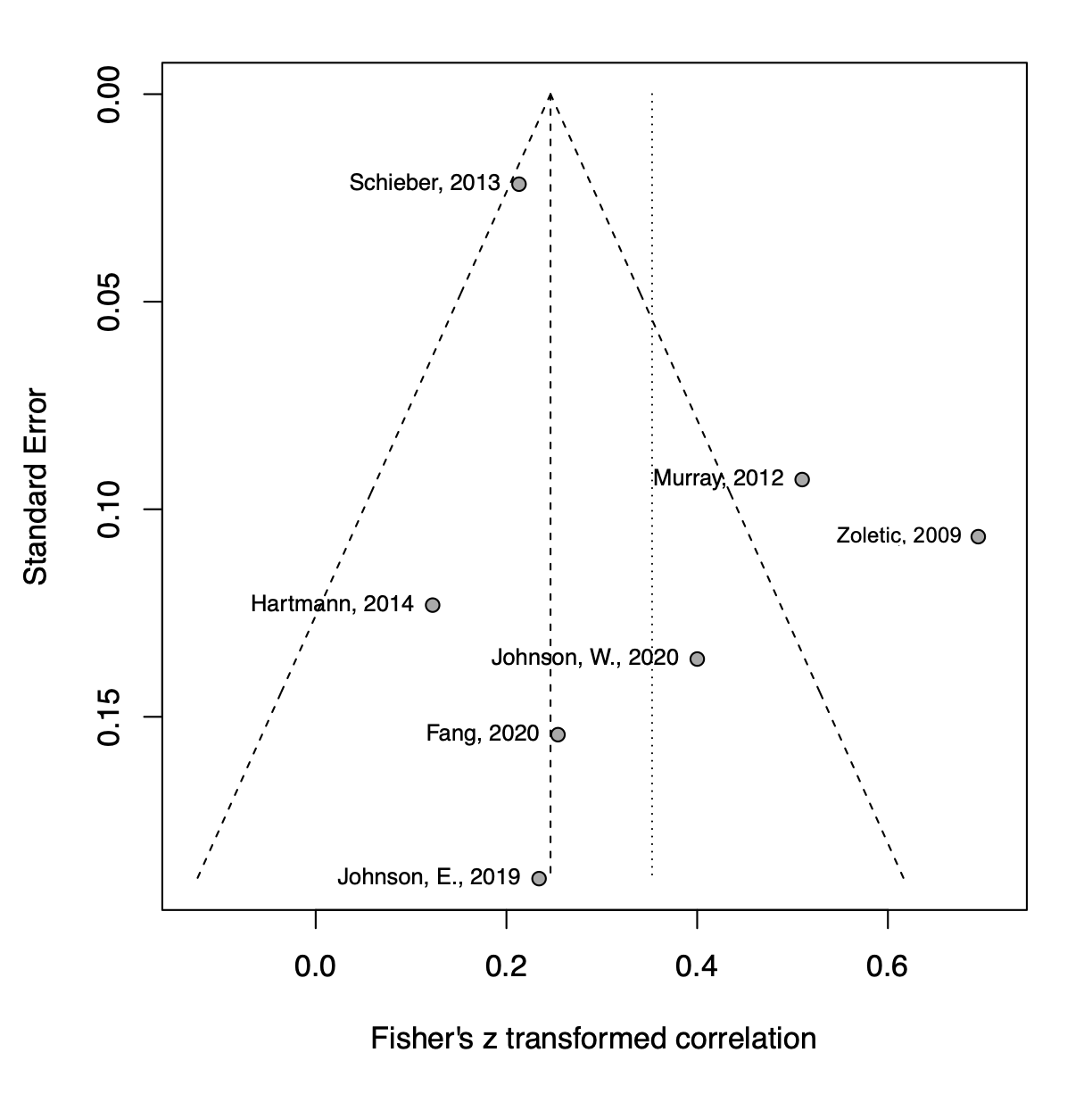
**

*Figure S7.* Funnel plot of publication bias assessment for secondary meta-analysis.

Note. Funnel plot illustrating potential publication bias in the secondary meta-analysis of the association between BDD symptom severity and perfectionism. Circles represent individual study effect sizes (Fisher’s z transformed correlations) plotted against their standard errors. The dashed lines indicate the pseudo 95% confidence limits around the pooled effect estimate. To enhance clarity and readability, study labels in the funnel plot were abbreviated to the first author’s last name followed by the publication year (author, year).
